# A method for combining multiple-units readout of optogenetic control with natural stimulation-evoked eyeblink conditioning in freely-moving mice

**DOI:** 10.1038/s41598-018-37885-w

**Published:** 2019-02-12

**Authors:** Jie Zhang, Kai-Yuan Zhang, Li-Bin Zhang, Wei-Wei Zhang, Hua Feng, Zhong-Xiang Yao, Bo Hu, Hao Chen

**Affiliations:** 1 Department of Physiology, College of Basic Medical Sciences, Army Medical University, Chongqing, 400038 P.R. China; 2 Department of Neurosurgery, Southwestern Hospital, Army Medical University, Chongqing, 400038 P.R. China; 3 Department 3, Institute of Surgery Research, Daping Hospital, Army Medical University, Chongqing, 400042 P.R. China

## Abstract

A growing pool of transgenic mice expressing Cre-recombinases, together with Cre-dependent opsin viruses, provide good tools to manipulate specific neural circuits related to eyeblink conditioning (EBC). However, currently available methods do not enable to get fast and precise readout of optogenetic control when the freely-moving mice are receiving EBC training. In the current study, we describe a laser diode (LD)-optical fiber (OF)-Tetrode assembly that allows for simultaneous multiple units recording and optical stimulation. Since the numbers of various cables that require to be connected are minimized, the LD-OF-Tetrode assembly can be combined with CS-US delivery apparatus for revealing the effects of optical stimulation on EBC in freely- moving mice. Moreover, this combination of techniques can be utilized to optogenetically intervene in hippocampal neuronal activities during the post-conditioning sleep in a closed-loop manner. This novel device thus enhances our ability to explore how specific neuronal assembly contributes to associative motor memory *in vivo*.

## Introduction

Eyeblink conditioning (EBC) is a commonly-used behavioral model for exploring the neural basis of associative motor learning^1,2^. In EBC, a conditioned stimulus (CS; e.g., tone or light) is always followed by an unconditioned stimulus (US; e.g., periobicular electric shock or corneal airpuff) that elicits a reflexive blink. To date, the underpins of EBC have been examined across various species, including rabbits^3–5^, rats^6,7^, mice^8–10^, cats^11,12^, guinea pigs^13,14^ and ferrets^15^. Among these species, rabbits were demonstrated to be very suitable for EBC researches because of their tolerance of restraint and large eye with few spontaneous eyelid movements^3–5^. However, mice begin to gain popularity because of their advantage in genetic manipulation^16^. In particular, recent combination of Cre-recombinase driver lines mice with Cre-dependent opsin viruses opens up an avenue to precisely manipulate targeted neural circuits, and thus provides good tools for exploring causal relationship between specific neural activity and EBC behavior^8,10,17^.

A critical step for optogenetic manipulation during EBC is to understand the effects of light on neural circuits *in vivo*. One ideal way to obtain readout of the light effect is combining multiple units recording (MUR) with optical hardware^18,19^. Although no free body movement is necessary for the EBC acquisition in mice^9,10,20^, recent study highlighted modulation of locomotor activity on the learning rate^8^. Also, free body movement contributes to relieve stress, which is one of the important factors that influence the EBC acquisition^21–23^. In particular, recent study has suggested that freely-moving can provide good opportunity to dissociate spatial (e.g. place) and non-spatial (e.g., CS-US association) encoding during EBC^24^. Consequently, it will be ideal for mice to freely move when they are receiving EBC training. However, combination of the freely-moving mouse preparation with optogenetic manipulation and MUR in the EBC experiments remains to be challenging.

One challenge is the limited size and weight of various MUR and optical implants that can be carried by the mice^18,19^. Moreover, technology for EBC in freely-moving mice includes delivery of both the CS and the US^20,25^. It turns out that tangle of various cables, optical fibers and delivery pipe may cause serious tension, which impedes free body movement of the mice. Therefore, the other challenge for freely-moving mouse preparation is adapting CS-US delivery to MUR-optical implants without obvious cable tangle.

In the current study, we describe a laser diode-optical fiber-Tetrode assembly, which could be combined with the delivery of blue light flash CS and airpuff US in the freely-moving mice. We found that this combination of techniques was suitable for revealing the effects of optogenetic manipulation in the freely-moving mice receiving EBC training. Furthermore, this apparatus could be utilized to perform on-line optogenetic intervention of hippocampal neural activities during the post-EBC training sleep.

## Materials and Methods

### Subjects

10 male C57BL/6 mice expressing light-activated inhibitory proton pump (archaerhodopsin from Halorubrum strain TP009, ArchT^26^) in the pyramidal cells of hippocampal CA1 were used in this study. The mice were 3–5 months old before the surgery. To achieve selective expression of ArchT, we crossed homozygous CamkIIa-cre mice from the Tg29-1 strain with homozygous mice from the Ai40D strain harboring a CAG-flox-Stop-Flox-ss-ArchT-EGFP. Both strains were purchased from the Jackson laboratory (Jax no. 005359 and 021188). The bred mice were CamkIIa::Ai40D (CamkIIa-ArchT) with fusion protein EGFP. Before the experiment and between the conditioning sessions, the mice were individually housed in a 12-hr light-dark cycle with lights on at 08:00, and had free access to food and water. All the experiments were conducted during the light phase of the cycle. All the experimental procedures were approved by the Animal Care Committee of Army Medical University (AMU) and were performed in accordance with the principles outlined in the NIH Guide for the Care and Use of Laboratory Animals. Efforts had been made to minimize the animals’ suffering.

### Construction of Laser diode-Optical fiber-Tetrode assembly

**Figure 1:**
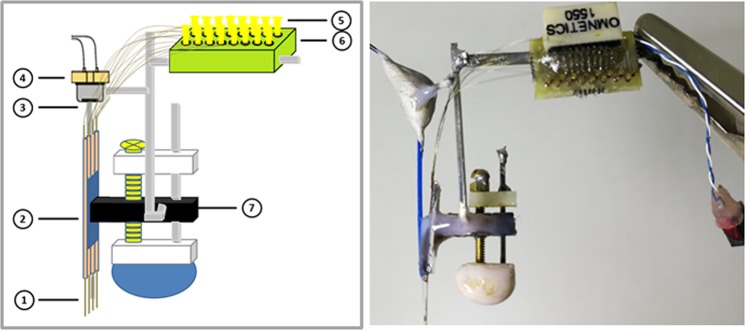
LD-OF-tetrode assembly for optogenetic control in freely-moving mice. *Left*: Schematic drawing of a homemade microdrive equipped with the LD-OF-tetrode assembly. Each LD-OF-tetrode assembly includes: ① 4 tungsten wire tetrodes, ② 4 polymicro pipes, ③ 1 optical fiber, ④ 1 green laser diode, ⑤ 16 gold pins, ⑥ 1 PCB board, and ⑦ 1 microdrive. The tip of optical fiber was attached ~500 um above the tips of tetrodes. Right: Picture of the LD-OF -tetrode assembly depicted in the left panel. Care should be taken to keep a 5-degree (or less) angle between the optical fiber and the tetrodes.

The Laser diode (LD)-Optical fiber (OF)-Tetrode assembly consists of four tetrodes, a green laser diode, an optical fiber and a home-made microdrive (Fig. 1). The materials for assembly construction are summarized in Table 1, and the assembly is constructed as follows.

**Table 1:** List of the parts of the apparatus combining multiple-units readout of optogenetic control with natural stimulation-evoked eyeblink conditioning in freely-moving mice.

Label	Item	Quantity	Supplier	Part number
**LD-OF-Tetrode**				
1	16-channel neuroconnector	1	Omnetics	A79016-001
2	Gold pins for electrical connection	16	Neuralynx	EIB Pins Small 1000
3	Quartz tubes	4	Polymicro Technologies	TSP100170
4	Microdrive	1	N/A	See refs^19,27^
5	Tungsten wires for extracellular recording	4	California Fine Wire	CFW2002936.
6	Litz Wire for signal transmission	2	Surplus Sales	LW-12/36
7	Laser diode for optogenetics	1	Osram	PL520B
8	Optical fiber	1	Thorlabs	FT200EMT
9	Silver paint for electrical shielding	1	M.E. Taylor Engineering	SP60+
10	SIP socket connectors for LD control	1	Mill-Max	853-93-100-10-001000
**TEBC training**				
1	Blue LED for CS	1	Avago Technologies	HLMP-AB74-WXBDD
2	Electrodes for orbicularis EMG recording	2	A-M Systems	791000
3	Needle and tubing for airpuff delivery	1	KDL	Infusion-18G
4	Copper mesh for headstage protection	4	Dexmet	3CU6-050FA
5	Connectors for airpuff delivery pipe holder,LED holder, and LED power supplier	3	Mill-Max	853-93-100-10-001000

### Laser diode-Optical fiber (LD-OF) coupling

The first step in constructing the LD-OF-Tetrode assembly is to couple a laser diode with an optical fiber. The procedures for LD-OF coupling are described as follows:

1. A 520-nm wave length green laser diode (PL520B, Osram, Germany) was quickly soldered a paired of lightweight wires to its anode and cathode connections. The other ends of the wires were then soldered to a SIP socket connector (Mill-Max, USA).
2. One tip end of an optical fiber was coupled to the prewired laser diode with the help of two manipulators (Newton, USA, Supplementary Fig. S1). This (proximal) tip end was manually moved close to the laser diode to obtain maximum light intensity on the other (distal) tip end. The green light emitted from the distal tip end of the optical fiber was measured by a light sensor (S130A photodiode, Thorlabs, USA). On average, the LD-OF couple efficient reached 30–50% if a 200-μm diameter fiber was used. When driven by a calibrated current source (LDC-205C, Thorlabs), these diodes could yield green light at the intensity of 15–25 mW/mm^2^. A drop of UV-curable optical glue (NOA-61, Norland Products, USA) was placed on the LD-OF interface. This glue was cured by UV light for at least 20 minutes. The coupling interface was further fortified by the grip cement (KeyStone Industries, USA). Afterwards, the coupled LD-OF was wrapped by conductive silver paint (M.E. Taylor Engineering, USA) to reduce electromagnetic artifacts generated by the laser diode (Supplementary Fig. S1B).

### Assembly of LD-OF, tetrodes and microdrive

The second step for constructing the LD-OF-Tetrode assembly is combining the coupled LD- OF with four movable tetrodes.

1. A neuroconnector (A79016-001, Omnetics, USA) was soldered to a PCB board (11.5 mm × 6.0 mm) with 18 holes (16 holes for neural signals, 1 hole for Reference, and 1 hole of Ground, Fig. 1). This connection was covered by Epoxy. A stainless steel bar was soldered on the back of PCB board. The neuroconnector-PCB board was mounted onto a homemade microdrive as illustrated in Fig. 1 and Supplementary Fig. S1C.
2. Each tetrode was made of 4 twisted tungsten wires of 20-μm in diameter (California Fine Wire, CFW2002936, USA). One end of the tetrode wire was inserted into the hole of neuroconnector-PCB board. The neuroconnector was electrically connected with the tetrodes by pressing the EIB small gold pins (Neuralynx, USA) into the corresponding holes. The other ends of tetrodes were inserted into 4 quartz tubes (TSP100170, Polymicro Technologies, USA), which were aligned in parallel (Fig. 1). Afterwards, the tetrodes were secured to the quartz tubes by using the surface intensive gel (Loctite 454 Prism, USA). The tetrode tips were 170-μm in space separation, and their impedances were measured (200–400 kΩ at 1 kHz).
3. With the help of 2 manipulators, the LD-OF was attached to the tetrodes so that its tip was 500 μm above the end of the tetrodes (Fig. 1). Care should be taken to keep a 5-degree (or less) angle between the optical fiber and the tetrodes (Fig. 1 and Supplementary Fig. S1C). A drop of UV-curable optical glue (NOA-61, Norland, USA) was placed on the fiber-tetrode interface. Again, this glue was cured by UV light for 20 minutes. Epoxy was applied to secure interconnection between the optical fiber and the tetrodes. By using epoxy, the laser diode was secured to the microdrive with a short stainless steel bar (Fig. 1 and Supplementary Fig. S1C).

### Surgery

**Figure 2:**
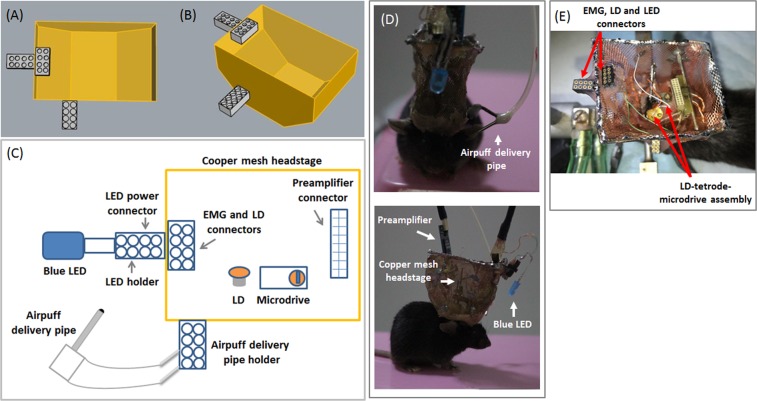
Combination of MUR and optical implants with CS-US delivery in freely-moving mice. (**A**) Top and (**B**) side view of the schematic diagram illustrating the copper mesh wall. A total of 3 SIP socket connectors were tethered to this wall. (**C**) Schematic diagram of various assembled components within the headstage. The description of various parts is listed in Table 1. (**D**) Front (upper) and side (bottom) view photograph of the assembly headstage in a freely-moving mouse. (**E**) Top view photograph of the assembled headstage including various SIP socket connectors and LD-OF-Tetrode assembly.

Mice were anesthetized with Isoflurane (0.6–1.0% by volume in O_2_) and kept on a heating pad to maintain body temperature. A local anesthesia was applied by hypodermically injecting 0.05 mL bupivacaine (0.025 mg/mL saline) along the scalp midline. To fully expose the skull above the cortex and cerebellum, a midline incision was made from Bregma to posterior border of the skull, and the underlying fascia was cleared with forceps and cotton swabs. Two small stainless- steel screws were placed in the skull above the cerebellum to function as ground and reference, respectively. Two stainless-steel wires (Bare diameter: 76 μm, Insulated diameter: 140 μm, Cat no. 791000, A-M Systems, USA) were subcutaneously implanted into the left upper orbicularis oculi muscle. These stainless steel wires and screws were secured on the skull using Metabond cement (Parkell, Japan), which was shaped to form a square wall. 4 triangle pieces of copper mesh (Dexmet, USA) were fixed on the top of Metabond walls with grip cement. For one group of mice (*n* = 4), the craniotomy was then made in the bone above the hippocampus. The LD-OF-Tetrode assembly was implanted perpendicularly to the midline at the following coordinates: AP: −1.9 mm, and ML: +1.6 mm (right hemisphere). During surgery, the tips of tetrodes were lowered into the neocortex area (Depth: 800 μm) with the help of stereotaxic arm (RWD, China). For the other group of mice (*n* = 5), an array of tetrodes was implanted into cerebellar cortex at the following coordinates: AP: −6.3 mm, ML: +1.4 mm, DV: 1.3 mm (left hemisphere). For one mouse (*n* = 1), a LD-OF-Tetrode assembly was implanted into the mPFC at the following coordinates: AP: +1.7 mm, ML: +0.5 mm, and DV: 1.0 mm (right hemisphere). The bottom part of microdrive was secured to previously-built square Metabond wall with grip cement. Low viscosity silicone (Kwik-Cast^TM^, WPI, USA) was applied to cover the craniotomy. The copper mesh was then folded upwards, and molded into a ‘square hat’ (Fig. 2). SIP socket connectors were secured to the copper mesh. The bottom mesh was strengthened by applying grip cement holding the skin apart. At the end of surgery, the jugular skin was sutured with thread to protect the unwounded area. After surgery, the mice were injected with 0.05 ml Buprenex intramuscular. During 5-days recovery, the LD-OF- Tetrode assembly was moved (70 μm/day) until it reached the CA1 pyramidal cell layer of dorsal hippocampus, characterized by large-amplitude SWR (150–250 Hz)^27,28^. The array of tetrodes in the cerebellar cortex was also daily moved until Purkinje cell firing was monitored. In addition, the mice were adapted to be handled by the researchers, and their weight was daily measured.

### Eyeblink conditioning

Following the postoperative recovery, the mice were adapted to the experimental environment for 2 days, 5 hours per day. During daily adaptation, the mice were allowed freely move in a containing box (45 cm × 25 cm × 20 cm) with the headstage connected to a 16-channel preamplifier (C3334, Intan technonlogies, USA) on the animal’s head. Moreover, the mice were habituated to plug-in and plug-out procedures to ensure that they could behave naturally. The containing box was located in a sound- and light-attenuating chamber. No stimuli were given during the habituation.

The adapted animals were given training of trace-paradigm EBC (TEBC) as previously described^29^. The CSs were LED pulses of blue light (150 ms in duration), and the USs were 100-ms airpuff directed to the left corneal via a blunted 27 gauge needle placed 5 mm from the mouse’s eye (Fig. 2). During TEBC, the CS offset was separated by a 250-ms trace interval from the US onset. Daily conditioning training consisted of 100 CS-US pairing trials. The intertrial interval varied from 18 to 28 s with a mean value of 23 s. The mice were trained for 5 consecutive days. Before and after daily conditioning training, the mice were returned to its home cage and allowed to sleep for at least 1 hr.

### Data acquisition

Within a given TEBC training day, multiple unit signals were continuously recorded across 3 stages (Pre-training sleep, TEBC, and Post-training sleep). As we recently reported^30^, the signals were amplified, digitized at 20 kHz using a preamplifier (C3334, Intan technonlogies) connected to the Intan interface board (RHD2000, Intan Technologies), and stored for off-line analysis in a 16-bit resolution format. The differentiated EMG signals were amplified (X 1000) and band-pass filtered between 150 and 1000 Hz. Together with those markers of applied stimuli (including the CS, US and optogenetic light stimulation pulse), EMG signals were fed into the digital input ports of data acquisition system (RHD2000, Intan Technologies). Data were visualized using NeuroScope (http://neurosuite.sourceforge.net)^31^.

### Laser diode illumination

#### CS-triggered illumination

Optical stimulation was applied through our LD-OF-Tetrode assembly. For optical stimulation of the dorsal hippocampus in the ArchT-expressing mice, the green laser diode was activated by the pulse currents (120–150 mA) from a laser diode controller (LDC-205C, Thorlabs), which was controlled by a programmable pulse generator (Pulse Pal v2, Sanworks, USA)^32^. The 400-ms pulse of light stimulation was triggered by the onset of the CS during TEBC. A power intensity of 15–25 mW/mm^2^ at the tip of optical fiber was measured before the surgery. This power intensity was postulated to be identical to that *in vivo*.

#### SWR-triggered illumination

In the on-line optogenetic manipulation experiment, local filed potential (LFP) was monitored during the NREM sleep period. A single channel from the middle of CA1 pyramidal cell layer with the largest amplitude SWR was selected for real-time processing of LFP. The post-TEBC sleep was separated into 2 halves. In the first half of post-training sleep (~30 min), the occurrence of SWR was continuously monitored, which did not trigger the green light stimulation. In the second half of post-training sleep (~30 min), a 100-ms current was triggered by each detected hippocampal SWR, which in turn activated the green laser diode. The SWR was detected using the sequential detection algorithm toolbox (http://fmatoolbox.sourceforge.net) as follows:

1. Band-pass filtering at 150–250 Hz (SWR) and signal rectification (Butterworth 3rd order);
2. Detection of events where the filtered envelope was twice higher than s.d. of filtered traces;
3. Selection of envelopes with peak signal 5 times higher than s.d.;
4. Selection of events with duration longer than 20 ms;
5. Elimination of events that were common to the SWR channel and a selected noise channel (i.e., an EMG electrode).

### Data analysis

#### Eyeblinks

The EMG activity during each trial was collected, rectified, and integrated with a 1-ms time constant using the algorithm introduced by Knuttinen *et al*.^33^. For each CS-US paired presentation trial, we first quantified the EMG activity for the baseline (1–300 ms before the CS onset) period across daily 100 trials. The EMG amplitude values for the 300-ms baseline period were averaged, and the standard deviation (s.d.) was calculated. The average value plus 4 times of the s.d. was defined as the threshold. Each trial with the maximum baseline EMG amplitude exceeding the threshold was defined as an invalid trial. The average number of invalid trials in a 100-trial single session across five daily acquisition sessions was [21.5 ± 3.2].

In this study, the startle eyeblink response (SR; 1–50 ms after the CS onset), conditioned eyeblink response (CR; 51–400 ms after the CS onset), and unconditioned eyeblink response (UR; 1–200 ms after the US onset) were exclusively measured in the valid trials. A significant eyeblink response was defined to exceed the average baseline by 4 times s.d. of the baseline activity for a minimal 25-ms duration. Any significant eyeblink response during the periods mentioned above was counted as a SR, a CR or an UR, respectively.

#### Unit clustering

The detailed procedures for single unit clustering have been described recently^27^. In brief, spikes were extracted from the high-pass filtered signals off-line, the waveforms were projected onto a common basis obtained by principal component analysis (PCA) of the data, and sorted into putative single units automatically using open-source software KlustaKwik^34^ followed by manual adjustment using the software Klusters (http://neurosuite.sourceforge.net/)^31^.

#### Optogenetic suppression of excitatory cells during TEBC

The response of isolated single unit during TEBC was tested with a series of 400-ms green light pulses triggered by the onset of the CSs. For comparison among all recorded units, the firing rates were normalized to their peak firing rates during a 2-s time window (1-s before and 1-s after the CS onset). This response mapping procedure allowed us to compare the firing rate of each unit before (1-s before each CS onset) and during the green light pulses (400-ms intervals starting from each CS onset). A unit was considered to be light-suppressed when the firing rate during the green light stimulation was significantly reduced as compared to the baseline activity.

#### Optogenetic suppression of excitatory cells during the post-TEBC sleep

For each isolated single unit, the spikes observed during each SWR trial were assigned to 10 ms time bins, beginning 400 ms before the SWR onset and extending 400 ms after the SWR onset. The firing rates (FR) for each bin across all SWR trials were calculated. The mean FR observed for the 40 bins before the SWR onset was used as the baseline activity, and their standard deviations (s.d.) were computed. The firing rate of each bin was then normalized by using Z score as follows (#bin refers to arbitrary time bin):

$${\rm{Z}}=({{\rm{FR}}}_{\#\mathrm{bin}}-{\rm{mean}}\,{{\rm{FR}}}_{{\rm{baseline}}})/{\rm{s}}{\rm{.d}}{\rm{.}}({{\rm{FR}}}_{{\rm{baseline}}})$$

### Histology

To visualize the tip position of LD-OF-tetrode in the hippocampus or cerebellum, electrolytic lesions (30 μA for 10 s, DC currents) were made at the end of all recording and optogenetic experiments. At 48 h after the electrolytic lesion, the mice were anesthetized with pentobarbital (100 mg/kg intraperitoneal) and perfused with saline and 4% paraformaldehydes (PFA; prepared in 0.1 M of phosphate buffer, pH 7.4). The brain was removed and post-fixed in 4% PFA for 24 hrs. Afterwards, the tissue was transferred to 30% sucrose solution for 48 hrs. Coronal sections with 20 μm in thickness were cut on a freezing microtome (CM1900, Leica, Germany) and collected in phosphate buffer saline (PBS, 0.01 M, pH 7.4).

Coronal slices underwent 3 wash steps of 10 min each in 0.01 M PBS, followed by mounting medium with DAPI fluorescence (F6057, Sigma) and cover slipping on the microscope slides. The extent of ArchT expression and tip placements of tetrodes were carefully checked and the images were acquired using a fluorescence microscope (BX53, Olympus, Japan).

### Statistics

Data are expressed as the mean ± S.E.M. unless otherwise noted. The statistical significance for behavioral analysis was determined by a one-way ANOVA with repeated measures and Tukey *post hoc* test using SPSS for the Windows package (v 13.0). Effects of optogenetic stimulation on hippocampal neuronal activities and LFP power spectrum were determined by Wilcoxon signed- rank test (2-tailed). A value of *p* < 0.05 was considered significant for all tests.

## Results

### Validation of LD-OF-Tetrode assembly

Our purpose is to combine extracellular recording with optogenetic stimulation when the freely-moving mice are receiving TEBC training. Considering that our optogenetic experiment relies on the light delivery during TEBC, we implanted a LD-OF-Tetrode assembly (Fig. 1) into the mouse brain. During the post-operative recovery, depth of the tetrode tips was manipulated by turning the screw (~70 um/day) until dorsal hippocampal CA1 area was reached. The implanted assembly was protected by a copper mesh headstage (Fig. 2A,B). The mesh headstage was anchored with 3 SIP socket connectors, which allowed plug-in of the blue LED, LD power, EMG recording and airpuff delivery pipe sockets (Fig. 2C–E). The total weight of this headstage was ~5 g after the implantation (~20% of mouse body weight), including the weight of acrylic affixing. After post-operative recovery, we found that the mice could freely move when carrying the implanted device (Supplementary Moive 1).

Moreover, the neuronal signals emitted from the preamplifier were multiplexed so that only one 12-strands litz wire was required to transmit the neuronal signals (Fig. 2D). The other litz wire was utilized to supply the power of blue LED and laser diode (Fig. 2D). This design largely reduced the tangle of litz wires with airpuff delivery pipe (Supplementary Moive 1), thus ensuring the free movement of mice when they were receiving TEBC training.

### Acquisition of TEBC

**Figure 3:**
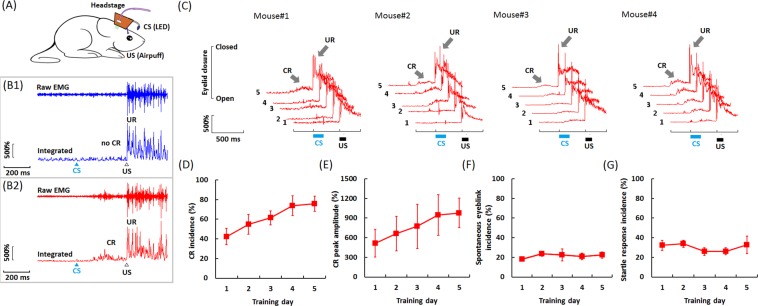
Acquisition of TEBC in freely-moving mice. (**A**) Schematic diagram of TEBC training in freely-moving mice. (**B**) Example of responses from a mouse in a non-CR (B1, blue) and CR (B2, red) trial, respectively. The CS was a 150-ms blue LED light (470 nm in wave length), while the US was a 100-ms airpuff to the cornea. A 250-ms time interval was inserted between the CS offset and the US onset. The top trace of each panel shows the raw EMG signal, whereas the bottom trace shows the rectified and integrated EMG signal. (**C**) Averaged eyelid responses of the valid CS-US presentation trials across five training days for each freely-moving mouse. (**D**) CR incidence and (**E**) CR peak amplitude measured from freely-moving mice (*n* = 4) across 5 conditioning training days. (**F**) Incidence of spontaneous eyeblink responses during the 300-ms baseline period. (**G**) Incidence of startle eyeblink responses (SR) in the 50-ms period after the CS onset. Data are shown as mean ± S.E.M. The CRs and URs were indicated by the arrows.

Next, we tested whether this device was suitable for TEBC training in freely-moving mice (Fig. 3A and Supplementary Moive 2). Figure 3B shows 2 representative examples of EMG activity from the upper eyelid of a freely-moving mouse. Examples are shown for a trial without CR (Fig. 3B, upper) and for a trial with CR (Fig. 3B, bottom), respectively. Note that all 4 mice established an association of the light CS with the airpuff US, as illustrated in Fig. 3C. We found that the mice exhibited a significant increase in the number of CRs across 5 training days (Fig. 3D; *n* = 4, *F*_[4,12]_ = 5.172; *p* = 0.012). On average, [350 ± 65] CS-US paired trials were required for the mice to reach the learning criteria. By the 5th day of TEBC training, the CR acquisition reached the asymptotic level (Incidence: [75.6 ± 7.7] %, Fig. 3D). Likewise, one-way ANOVA with training day as within- subject factor revealed significant increase in the CR peak amplitude (Fig. 3E; *F*_[4,12]_ = 35.879, *p* = 0.009). With respect to the timing of acquired CRs, the responses on the last day of training had a average peak time of [130 ± 30] ms before the US onset.

The CR performance is reported to be highly correlated with neuronal activities in the cerebellum^20^. To assess how the EMG signals we recorded are related to the eyelid movements, we compare temporal pattern between CS-evoked EMG signals with cerebellar cortical neuronal responses. It was found that Purkinje cells exhibited significantly decreased response to the CS when the CR was expressed (Supplementary Fig. S2C). Moreover, averaged population responses of Purkinje cells were resemble to the temporal pattern of learned CRs (Supplementary Fig. S2I) implying the cerebellum-related CR expression.

It should be noted that the CR incidences were exclusively evaluated in the valid trials without spontaneous eyeblink response during the baseline period. In this study, the mice emitted few spontaneous eyeblink responses in the baseline period, and showed no significant change across 5 training days (Fig. 3F; on average: [21.5 ± 3.2] %, *n* = 4, *F*_[4,12]_ = 0.374, *p* = 0.823). Additionally, the mice exhibited a low level of SR in the 50-ms period after the CS onset (Fig. 3G; on average: [30.3 ± 5.1] %, *n* = 4, One-way ANOVA with training day as within-subject factor: *F*_[4,12]_ = 0.929, *p* = 0.479).

### Multiple-units recording during TEBC

**Figure 4:**
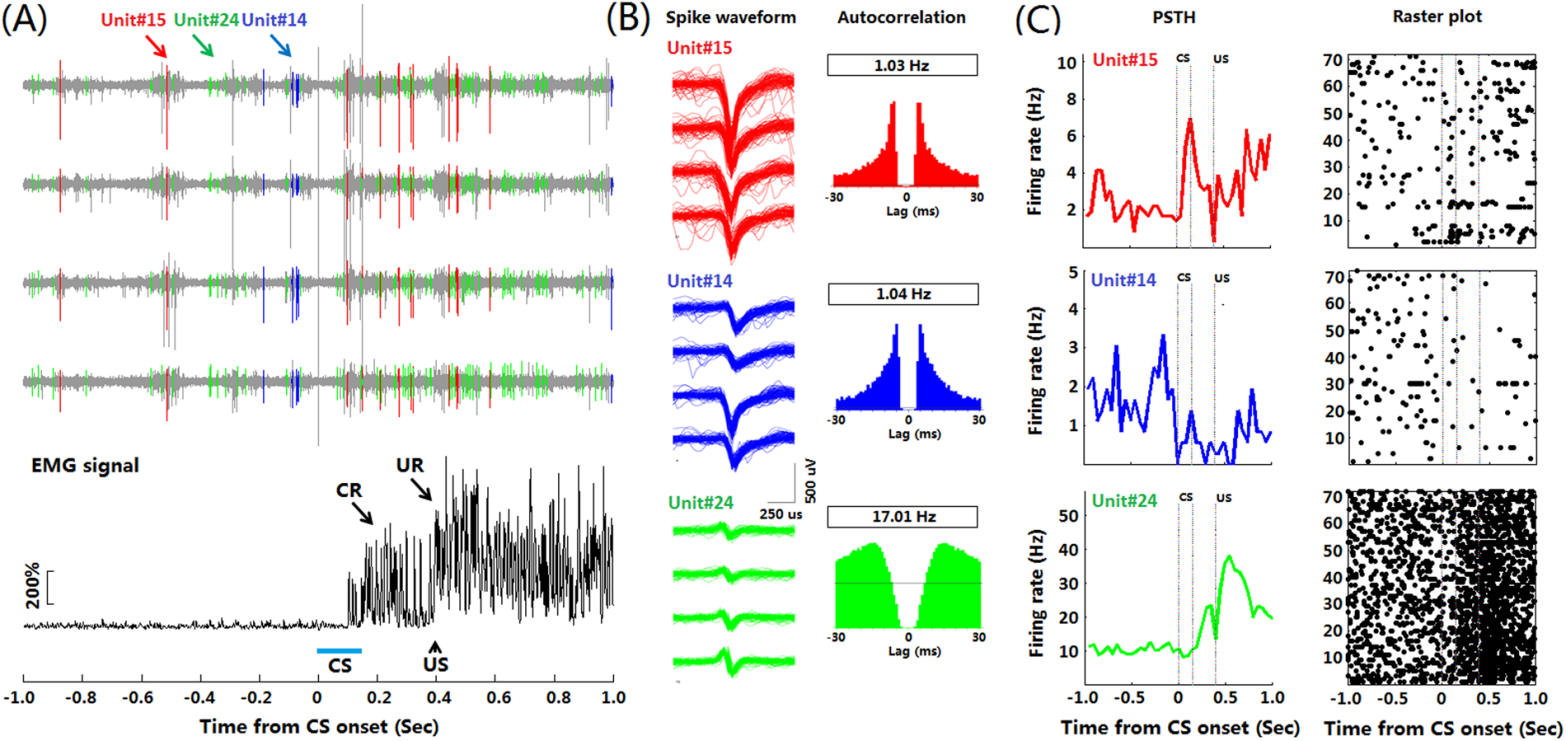
Multiple units recording in freely-moving mice during TEBC. (**A**) Representative hippocampal CA1 neuronal activities during TEBC in a freely-moving mouse. *Upper:* Firing activities of 3 representative isolated single units in a CS-US paired presentation trial. *Lower*: The rectified and integrated EMG signal for the same CS-US trial. (**B**) Spike waveforms (*Left*) and autocorrelograms (Right) of three single isolated units shown in (**A**). The spike waveforms on each recording channel of a tetrode are illustrated. Same isolated units and colors in (**A**,**B**). (**C**) Peri-stimulus histogram (PSTH, left) and raster plot (right) revealed the firing patterns of three isolated units illustrated in (**A**).

A critical step to obtain readout of optogenetic control is performing MUR in mice receiving TEBC training. To this end, we tested the utility of our LD-OF-Tetrode device for MUR in freely- moving mice. The *post hoc* histological results showed that all the recording sites were located in the dorsal hippocampal CA1. We recorded signals from hippocampal CA1 area across 3 epochs: pre-training sleep, TEBC training, and post-training sleep (Supplementary Fig. S3A). Figure 4 shows examples of three recorded hippocampal units during TEBC. High signal-to-noise ratio ensured accurate clustering of simultaneously recorded single units (Fig. 4B and Supplementary Fig. S4). PSTHs were computed to classify the recorded units into different groups, depending on their firing patterns associated with the CS presentation (Fig. 4C and Supplementary Fig. S5A). Analysis of recorded units in this mouse (*n* = 29) revealed that temporal pattern of hippocampal neuronal activities were resemble to that of the CR performance (Supplementary Fig. S5B).

In particular, neuronal signals during the presentation of US were not contaminated with EMG signals (Fig. 4A). This advantage allows us to determine whether single unit is US-responsive. To evaluate how stable these single units were, we also examined the spike waveform of single unit across three different epochs in a training day. It was found that the spike waveform in each channel remained stable over ~200 minutes (Supplementary Fig. S3B). This result indicated that our LD-OF-Tetrode assembly could be utilized to perform stable MUR in the freely-moving mice. Although MUR was stable in each recording session, however, tetrodes were manually moved to get maximum number of single units in each session. Consequently, the average and maximum duration of single neuron recordings were not examined across sessions.

### Readouts of optogenetic control during TEBC

We next tested the ability of our LD-OF-Tetrode assembly to obtain readouts of optogenetic control during TEBC (Supplementary Moive 3). The tip light intensity of each LD-OF-Tetrode was evaluated before implantation (Wave length: 520 nm, Duration: 400 ms, Intensity: 20 ± 2.1 mW/mm^2^). To determine the effective size of optogenetic inhibition, we implanted a modified LD-OF-Tetrode assembly, which contained 2 long tetrodes and 2 short tetrodes (Supplementary Fig. S6A). It was found that, in the tetrodes 1000 μm away from the tip of optic fiber, green light at the intensity of 5 mW/mm^2^ could not totally inhibit firing activities (Supplementary Fig. S6B,C), whereas green light at the intensity greater than 10 mW/mm^2^ totally inhibited neuronal activities in the mPFC. These results indicated that green light at the intensity greater than 10 mW/mm^2^ could be utilized to inhibit the firing activities recorded ~1.0 mm from the tip of optic fiber.

**Figure 5:**
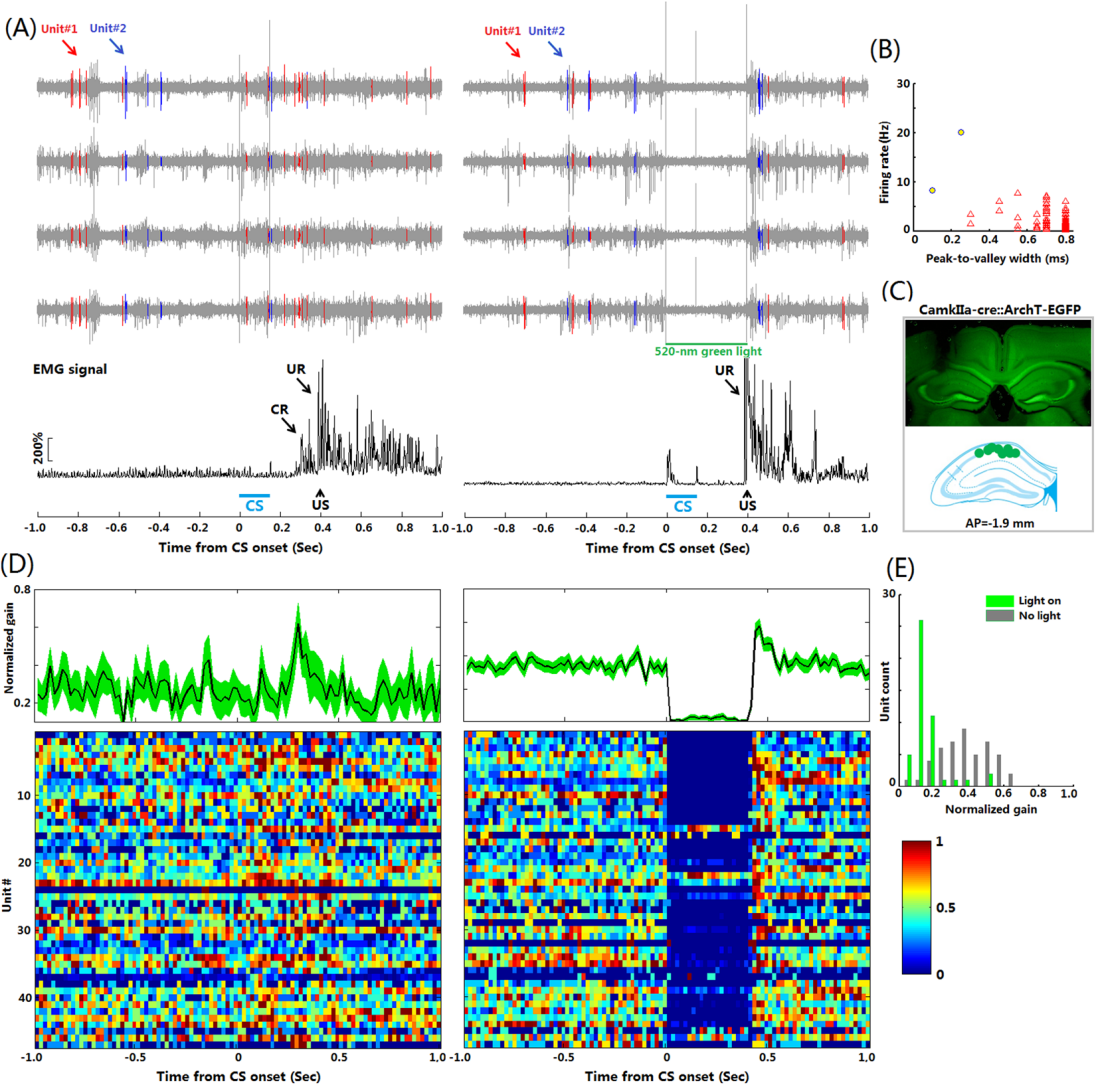
Readout of optogenetic intervention during TEBC. (**A**) Optogenetic inhibition of hippocampal PYR units during TEBC. *Upper*: (**A**) CS-US presentation trial without green light stimulation and a CS-US trial with 400-ms green light stimulation after CS onset. 2 representative isolated units are shown. They were obviously inhibited by the green light. *Lower*: Two rectified and integrated EMG signals for the same CS-US trials in the upper panel. (**B**) The recorded hippocampal units were classified into 2 groups: putative pyramidal cells (PYR, *n* = 47, triangles) and putative interneurons (*n* = 2, circles) based on their firing rates and spike waveform width. (**C**) Representative recording site in the hippocampus of CamkIIa-cre::ArchT- EGFP mouse. Schematic drawings of the valid recording sites in 4 mice are exhibited. (**D**) *Upper*: Normalized gain of spike activities across putative PYR units in the CS-US trials without and CS-US trials with green-light stimulation (*n* = 47). Shaded area represents S.E.M. *Lower*: The peri-CS firing activities were illustrated for each putative PYR unit before (left) and after (right) the optogenetic intervention. (**E**) Efficiency of the optogenetic inhibition at the population level (*n* = 47). 87% of the recorded putative PYR units were inhibited by the 400-ms green light pulses.

Next, we evaluated the readout of optogenetic control during TEBC. On the 6^th^ training day, the mice received 3 continuous recording epochs: pre-sleep training, sleep, and post-sleep training. The firing activities in the pre-sleep TEBC training were functioned as a control to exhibit how population hippocampal units response to the CSs (Fig. 5). In the current study, we isolated 49 hippocampal units from 8 tetrodes in 4 ArchT-expressing mice (Fig. 5A–C). Based on their firing rates and width of spike waveform, the recorded units were classified into 2 groups: putative pyramidal cells (PYR, *n* = 47) and putative interneurons (*n* = 2, Fig. 5B). In the pre-sleep training (Control) stage, the presentation of CSs evoked significant increases in the firing rates of PYR (Fig. 5A,D left panels, *n = *47, *Z* = 4.966, *p* < 0.001). In the post-sleep TEBC training, however, the increased PYR activities during the CS presentation were robustly inhibited by green light stimulation (Fig. 5A,D right panels, *n = *47, Z = −5.588, *p* < 0.001). The statistical analysis revealed that 87.2% (41/47) of the recorded PYR units were immediately inhibited by green light (firing rate decreased 30.9–100%, mean ± s.d. = [94.3 ± 13.8] %, *n* = 41). In contrast, the remaining 12.8% (6/47) of recorded PYR units increased their firing rate (firing rate increased 0.1–185.7%, mean ± s.d. = [60.5 ± 65.8] %, *n* = 6), possibly as a result of neural circuit effect. Therefore, these results suggested that our LD-OF-Tetrode device was capable of obtaining readout of optogenetic control during TEBC.

### Readouts of optogenetic control during post-TEBC sleep

**Figure 6:**
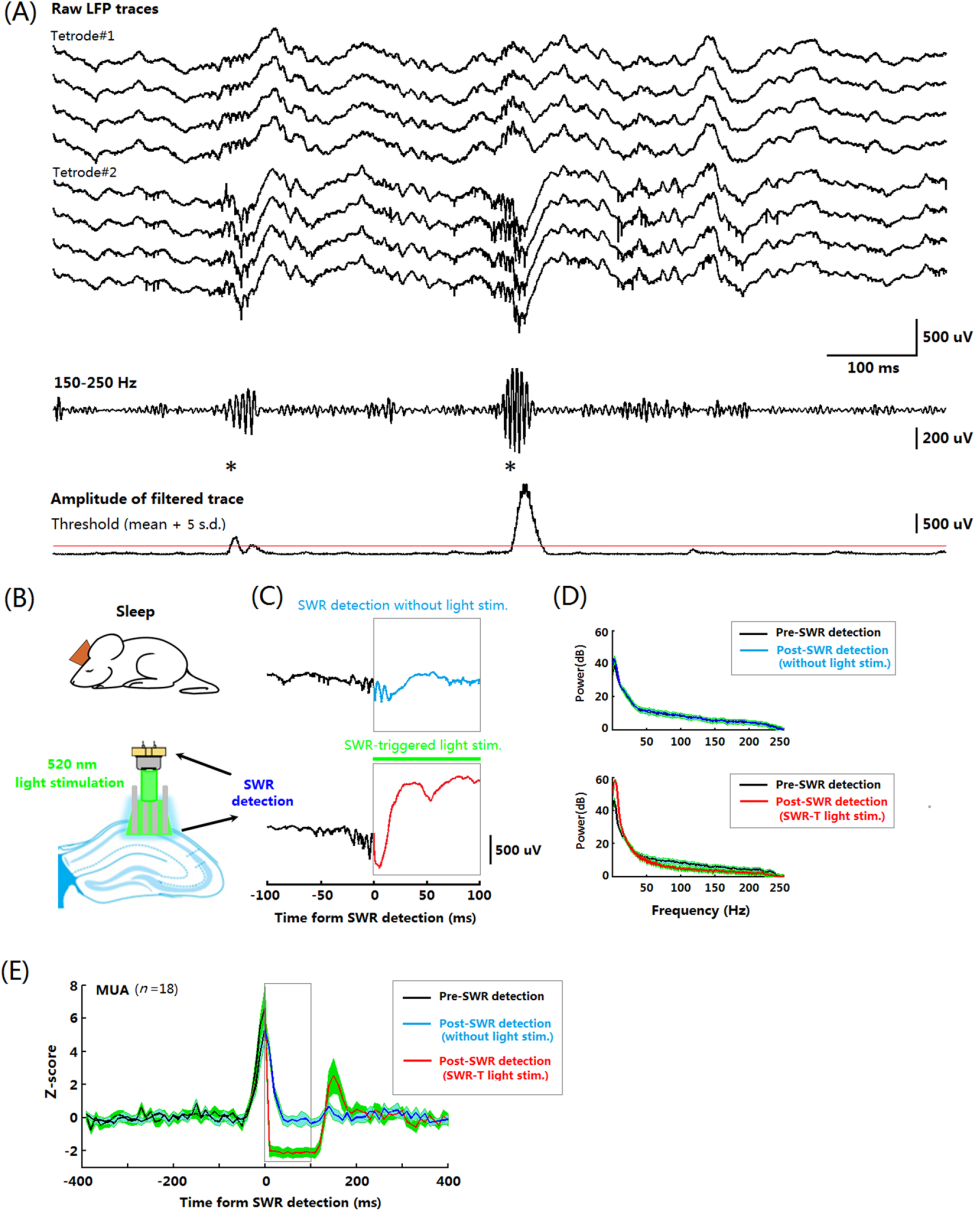
Real-time optogenetic silencing of putative PYR contingent upon SWR detection. (**A**) The threshold for real-time SWR detection was set to 5× s.d. above the mean power. Top, raw LFP traces recorded from 2 tetrodes in the dorsal hippocampal CA1 area. Middle, a trace filtered in a SWR-frequency (150–250 Hz) band; Bottom, a trace representing the LFP envelope amplitude. The asterisks indicate the detected SWR onsets. (**B**) Schematic diagram of real-time optogenetic experiments during the post-TEBC sleep. (**C**) Representative abolishment of the ongoing SWR by the optogenetic inhibition. (**D**) Power of LFP oscillations 100-ms before (black traces) and 100-ms after (red trace) the onset of green lights. It was revealed that LFP power in high-frequency band (150–250 Hz) oscillation was significantly decreased after the green light onset. (**E**) Population responses of putative PYRs (*n* = 18) before (black) and after (red) the green light onset.

The reactivation of stored representations during hippocampal SWR is believed to be one of the neural mechanisms underlying memory consolidation. We thus applied our device to real- time closed-loop stimulation experiments. In this case, we tested whether the LD-OF-Tetrode device could be utilized to optogenetically abolish the hippocampal SWR once detected. To this end, 2 mice were monitored on day 7. The onset of hippocampal SWR was detected on-line by filtering the LFP signals in ripple-band (150–250 Hz) during NREM sleep (Fig. 6A). The on-line detection rate was 85% and 90% of *post hoc* detected SWR in the two tested mice, respectively. In the first half of sleep (Control stage, ~30 min), the onset of SWR did not evoke light stimulation (Fig. 6B,C upper). In contrast, in the second half of sleep, the onset of SWR evoked 100-ms green light stimulation in the hippocampus (Fig. 6B,C bottom). The power of ripple-band oscillation was significantly decreased by green light stimulation (Fig. 6D, upper panel; *n* = 334, *Z* = −6.700, *p* < 0.001). Consistent with this finding, the hippocampal PYR activities were significantly inhibited by green light stimulation (Fig. 6E; *n* = 18, *Z* = −3.724, *p* < 0.001). These results suggested that our LD-OF-Tetrode device was capable of intervening in high-frequency neural oscillations.

## Discussion

In the present study, we adapted a LD-OF-Tetrode assembly to the CS-US delivery apparatus in freely-moving mice. Our results demonstrate that, with this novel device, the adaptive CRs are gradually acquired in the mice. Importantly, stable MUR can be performed to reveal inhibitory effect of light stimulation on the hippocampal PYRs when the freely-moving mice are receiving TEBC training. Moreover, this device could be used to optogenetically intervene in hippocampal SWRs in a closed-loop manner during the post-TEBC sleep.

Mice are demonstrated to be very suitable for optogenetic experiments due to its feasibility in genetic manipulation^18,35,36^. Recently, attempts have been made to utilize several lines of Cre-mice to allow fast and cell type-specific manipulation of neuronal activities^10,37,38^, which opens up an avenue to explore the mechanisms underlying EBC. However, the acquisition of EBC is demonstrated to be modulated by locomotor activity in mice^8^. Additionally, the mice are intolerant for restraint as well as the rabbits^3–5,18,25^. Therefore, it will be ideal if the mice can freely-move in the experimental arena when they are performing the EBC task. In our design, the blue LED and the airpuff delivery pipe were tethered to their adaptors on the copper mesh (Fig. 2). This design provided enough reliability for delivering the light flash CS and the corneal airpuff US in the freely-moving mice, irrespective of their heads’ direction and position. We found that the CRs were learned in all tested mice across 5 training days (Fig. 3), and the asymptotic learning rate was similar to that previously reported^20,29^. In the meanwhile, the learned CRs seemed to be adaptive since they mostly occurred in the 200-ms period before the US onset^39,40^. Moreover, temporal pattern of the learned CRs was highly related to that of Purkinje cells in the cerebellar cortex (Fig. S2). Taken together, these results thus suggest that our newly-designed device could be utilized to perform EBC training in freely-moving mice.

Up-to-date, combination of the optogenetic manipulation and MUR with the CS-US delivery of EBC in freely-moving mice remains to be challenging. One challenge is the limited weight of various MUR-optical implants and CS-US delivery apparatus that can be carried by the mice. To overcome the challenge, several laboratories modified a training apparatus in which the mice were head-fixed and running on a wheel^8,9,20,41^. This method helps to reduce weight burden of the animals’ head and neck in mice ongoing EBC training. The other challenge is tangle among various cable and airpuff delivery pipes. In the traditional design, a bundle of cables (16- or 32-ch) was required to transfer amplified multiple unit signals to the data acquisition system^42,43^. The thick cable bundle was easy to tangle with optic fiber and airpuff delivery pipe, which produced serious tension so as to impede the free movement of mice. Based on those previous EBC studies in mice^39,40,44,45^, we made improvement by further adapting a LD-OF-Tetrode assembly to EBC training apparatus. This combination of techniques allows us to get fast readout of optogenetic control in freely-moving mice with single-unit resolution (Fig. 5). In addition, it will be promising that the technical development can be utilized to explore the neural basis of more complex forms of associative learning tasks such as instrumental learning^46^ and operant reward learning^47^.

Several advantages of our combination technique help to successfully perform simultaneous optogenetic control and MUR when the freely-moving mice are receiving TEBC training. First, the weight of each LD-OF-Tetrode assembly was ~5 g, thus not placing too much burden upon the mice’ head and neck (Supplementary Movie 1). However, more efforts should be made to further reduce burden of the freely-moving mice. For example, 3D printed plastic material can be utilized to reduce weight of ‘square hat’ headstage. Second, on-head signal amplification and multiplexing allowed one cable for transferring the electrophysiological signals^19,48^, while the other cable for supplying the power of laser diode and blue LED (Fig. 2). Because both cables were thin and soft, our design thus robustly reduces tension caused by the cable tangle. Third, the microdrive was physically separated from the head preamplifier connector by flexible tungsten wires (Fig. 1). This design ensured that the MUR remained to be relatively stable when plugging and unplugging the preamplifier connector^48^. Fourth, relative to the commonly-used periorbital electrical shock as the US, the corneal airpuff was more natural, and able to reduce the animals’ uncomfortable responses^49^. Also, the utilization of corneal airpuff as the US could avoid affecting the EMG signal, which was easy to be saturated by the periorbital electrical shock US^50^. This advantage thus allows complete analysis of the US, thus facilitating examination of the effect of drugs or genetic manipulations on the expression of URs. Fifth, relative to the commonly-used auditory stimulus, a blue light flash (150 ms in duration) was utilized as the CS (Fig. 2). We showed the results that the freely-moving mice acquire trace CRs (Fig. 3). Likewise, recent studies have reported modified EBC training procedures in which the mice were head-fixed on a wheel. In these studies, the mice learned the association of blue light CS and airpuff US with a rate similar to our observation^8–10,20,41,45,51^.

In this study, our newly-deigned device has been demonstrated to be capable of manipulating hippocampal neuronal activity not only during TEBC, but also during the post TEBC-training sleep (Fig. 6). Evidence has accumulated that the acquisition of TEBC depends on the hippocampus^52–55^, whereas its consolidation relies on extra-hippocampal structures (i.e., medial prefrontal cortex (mPFC)^4,54–58^). In particular, the reorganization of TEBC memory trace has been revealed to occur in off-line post-TEBC training stage^56,57,59^. The post-learning sleep is thought to be an important off-line state, which plays critical role in the consolidation of memory^60,61^. With respect to the neural mechanisms underlying memory trace shift, information transfer from the hippocampus to the mPFC during the hippocampal SWR has been implicated^62,63^. As a result, rapid interruption of communication between the hippocampus and mPFC will open a door to addressing novel questions like how associative motor memory is consolidated in off-line state.

The current study, to our best knowledge, is first to describe a method for combining multiple -units readout of optogenetic control with natural stimulation-evoked EBC in freely-moving mice. This combination illustrates the usefulness of LD-OF-Tetrode assembly in time-sensitive behavioral paradigms in freely moving mice, thus being able to disentangle temporal and spatial influence on the neuronal firing of specific units in the brain^24^. Also, it will enhance our ability to examine associative motor learning-induced neuronal plasticity in specific neural circuits.

## Supplementary information

Moive-1

Moive-2

Moive-3

Supplmentary material
