# Supplementary material for: A method for combining multiple-units readout of optogenetic control with natural stimulation-evoked eyeblink conditioning in freely-moving mice
Source: Sci Rep. 2019 Feb 12;9:1857. doi: 10.1038/s41598-018-37885-w (PMC6372581; doi:10.1038/s41598-018-37885-w)
Supplement: Supplementary file 4 — Supplmentary material [file 41598_2018_37885_MOESM4_ESM.docx]

**Supporting Online Material for**

**A method for combining multiple-units readout of optogenetic control with natural stimulation-evoked eyeblink conditioning in freely-moving mice**

Jie Zhang, Kai-Yuan Zhang, Li-Bin Zhang, Wei-Wei Zhang, Hua Feng,

Zhong-Xiang Yao, Bo Hu, Hao Chen

To whom correspondence should be addressed:

E-mail: [haochen@tmmu.edu.cn](mailto:haochen@tmmu.edu.cn) (H. Chen) & [bohu@tmmu.edu.cn](mailto:bohu@tmmu.edu.cn) (B. Hu)

**This file includes 6 figures.**

**Suppl. Figure 1** Laser diode-optical fiber coupling, related to Figures 1 and 2.

**Suppl. Figure 2**  Correlation of CR performance with neuronal activities in the cerebellar

cortex.

**Suppl. Figure 3**  Stability of multiple-units recording in freely-moving mice, related to

Figure 4.

**Suppl. Figure 4** An overview, showing auto- and cross-correlograms of representative

isolated single units, related to Figure 4.

**Suppl. Figure5** Representative and population responses of hippocampal units during TEBC, related to Figure 4.

**Suppl. Figure6** Estimation of the effective size for optogenetic control, related to Figure 5.

**Figure_S1**


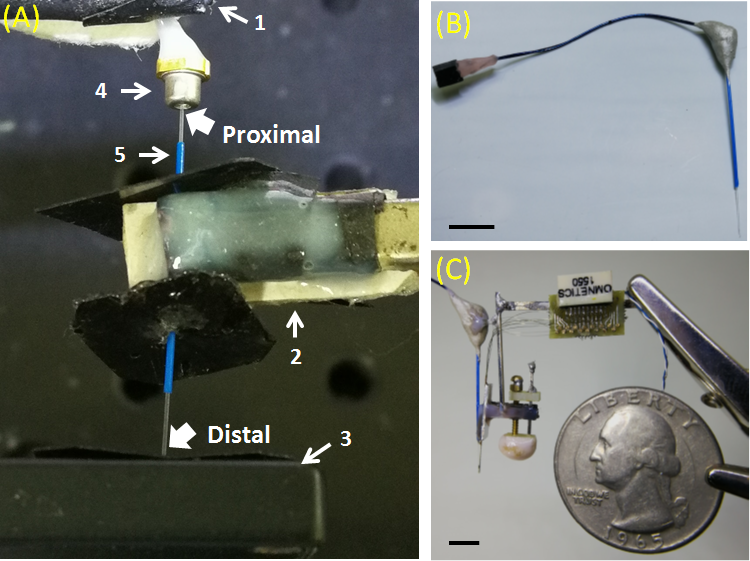


**Figure S1| Laser diode-optical fiber coupling. (A)** Alignment and coupling of laser diode and optical fiber. **1&2**: micromanipulators; **3**: Light sensor (S130A, Thorlabs); **4**: Green laser diode; **5**: optical fiber (~3.5 cm in length). Manipulator #1 holds the pre-wired laser diode. Manipulator #2 holds the optical fiber. 2 pieces of black paper with pinholes are slipped over the fiber and cover the light sensor aperture and diode. The sensor reading is 0 when the laser diode is not activated. The diode-fiber interface will be secured by the UV-curable glue when maximum light intensity in the distal tip of optical fiber is obtained. **(B)** The coupled diode-fiber interface is fortified by grip cement and afterwards wrapped by conductive silver paint. **(C)** Single coupled diode-fiber is further coupled to four movable tetrodes, which are then mounted on a homemade microdrive. Scale bars in (B) and (C) represent 5 mm.

**Figure_S2**

**
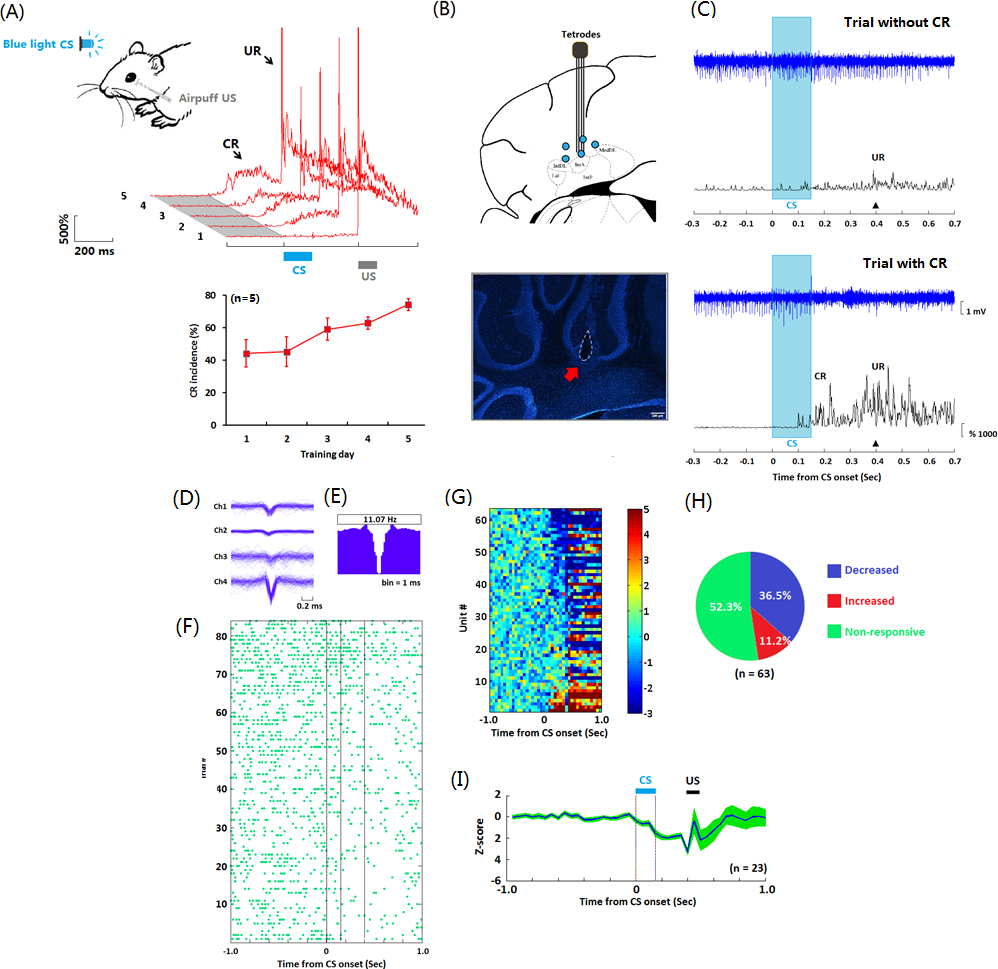
**

**Figure S2| Correlation of CR performance with neuronal activities in the cerebellar cortex. (A)** Acquisition of TEBC in freely moving mice (*n* = 5). **(B)** Recording sites in the cerebellar cortex. A representative recording site is indicated by red arrow. **(C)** Examples of cerebellar cortical neuronal responses from a non-CR (B1, upper) and CR (B2, bottom) trial, respectively. **(D)** Spike waveforms and **(E)** autocorrelogram of a representative cerebellar cortical unit. **(F)** Raster plot revealed firing pattern of the isolated cerebellar cortical unit illustrated in (D). **(G)** Population responses of cerebellar cortical units during TEBC training. Heatmap rows represent the Z-score- transformed PSTH for individual units (*n* = 63), and columns represent time bins relative to the CS onset (50-ms in width). **(H)** CS-responsive subpopulations in the cerebellar cortex. **(I)** Z-score- transformed average PSTH for CS-decreased responsive units (*n* = 23). It was shown that this population response is highly resembled to the performance of trace CRs.

**Figure_S3**


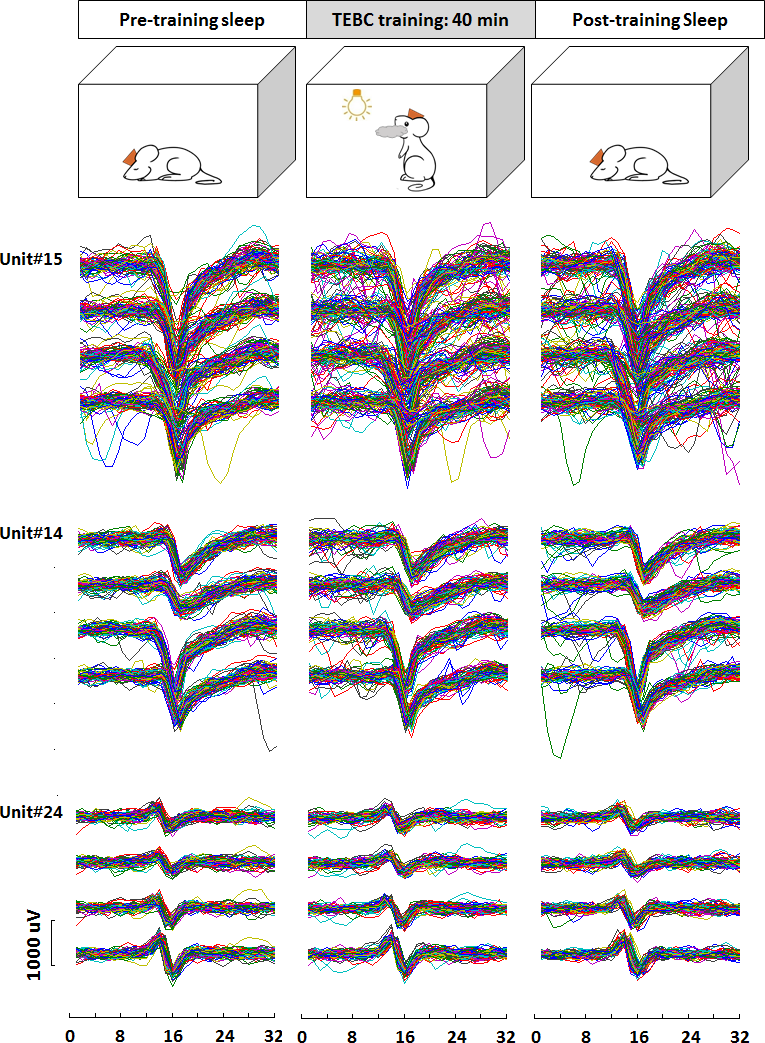


**Figure S3| Stability of multiple-units recording in the freely-moving mice. (A)** Three epochs constituting a daily recording session: pre-training sleep (~1 hr. in home cage), TEBC training (~ 40 min in experimental box), and post-training sleep (~1 hr. in home cage). **(B)** Superimposed spike waveforms (*n* = 100) of 3 isolated single units across three recording epochs. These units were illustrated in Figure 4. It was shown that spike waveforms of the isolated single units remained stable in each recording channel across three epochs.

**Figure_S4**


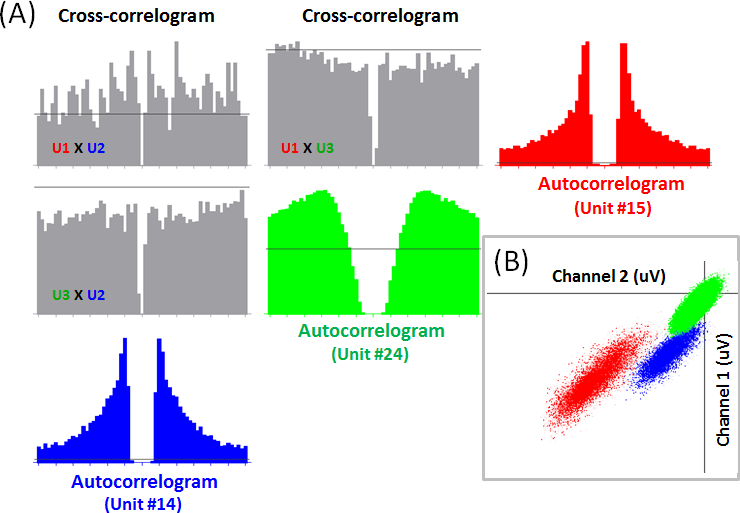


**Figure S4| An overview, showing auto- and cross-correlograms of representative isolated single units.** **(A)** Auto-(colored) and cross-correlation (gray) histograms were shown for 3 isolated single units illustrated in Figure 4. The autocorrelograms showed clear refractory period for each cluster, whereas the crosscorrelograms showed relatively symmetrical distribution for each pair of units. **(B)** A plot of amplitude of spikes on a pair of channels after spike sorting.

**Figure_S5**

**
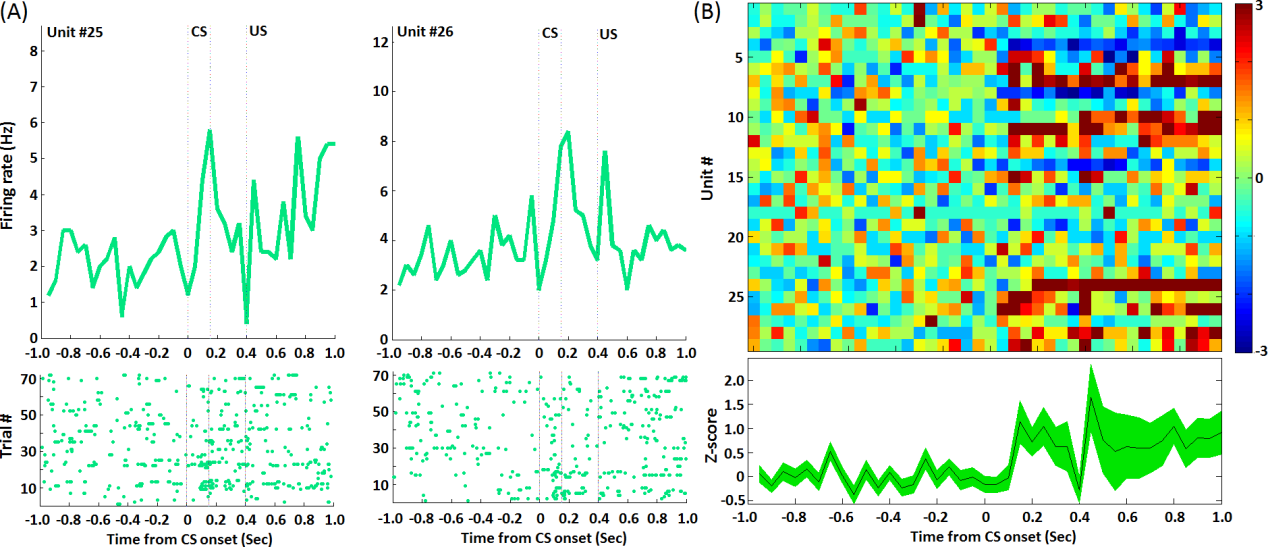
**

**Figure S5| Representative and population responses of hippocampal units during TEBC. (A)** Increased neuronal activities were evoked by the CS presentation in 2 representative units (#25 and #26, related to panel B). **(B)** Population responses of hippocampal units (*n* = 29) during TEBC training. It was shown that significantly increased responses were evoked by the CS presentation. In particular, the population responses are resembled to the performance of trace CRs (related to Figure 4, panel A, bottom).

**Figure_S6**


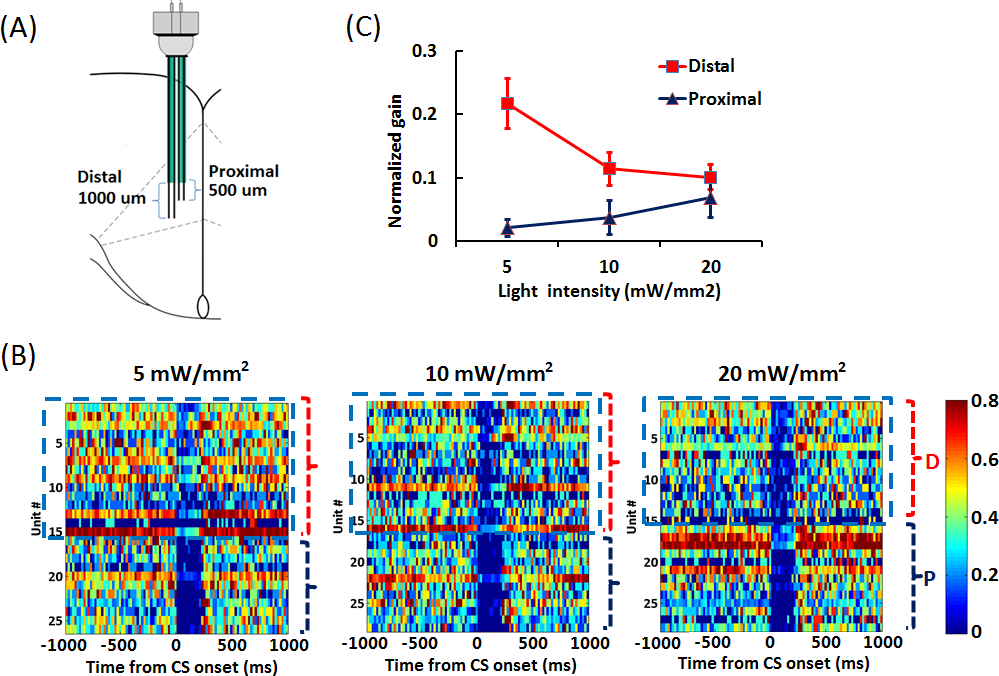


**Figure S6| Estimation for the effective size of optogenetic control. (A)** Schematic diagram of a modified LD-OF-Tetrode assembly. The assembly contains two groups of tetrodes with different length. The tips of one group of tetrodes (*n* = 2) were ~ 1000 um away from the tip of optic fiber, whereas tips of the other group of tetrodes (*n* = 2) were ~ 500 um away. This design allowed us to estimate the light diffusion in the brain. **(B)** Heatmap illustrates the effects of green light at different intensity on neuronal activities in the distal (D: indicated by rectangles) and proximal (P) tetrodes, respectively. **(C)** Statistical analysis revealed the effects of green lights on neuronal activities in the distal (red squares) and proximal (blue triangles) tetrodes. It was shown that the green light at the intensity greater than 10 mW/mm^2^ could be utilized to inhibit firing activities recorded ~ 1000 μm away from the tip of optic fiber.
